# Blended learning in medical education in India—a literature-based analysis and description

**DOI:** 10.3389/fmed.2026.1814270

**Published:** 2026-04-30

**Authors:** Ambuj Roy, Akanksha Pal Singh, Rashmi Ramachandran

**Affiliations:** 1 Department of Cardiology, All India Institute of Medical Sciences, New Delhi, India; 2 S.E.T (Skills, E-learning & Telemedicine) Facility, All India Institute of Medical Sciences, New Delhi, India; 3 Department of Anesthesiology, Pain Medicine and Critical Care, New Delhi, India

**Keywords:** blended learning, e-learning, flipped classroom, Indian medical education, teaching/learning strategies

## Abstract

**Background:**

Blended learning has gained momentum in Indian medical education and has the potential to improve access to knowledge and educational outcomes in underserved areas.

**Objective:**

This review sought to identify current BL models in Indian medical education, evaluate their impact on knowledge acquisition, engagement, and performance, and explore implementation barriers and enablers.

**Methodology:**

This structured narrative review used electronic databases such as PubMed, Scopus, and Google Scholar for studies on blended learning in Indian undergraduate and postgraduate medical education. Two independent reviewers screened titles, abstracts, and full texts, with disagreements resolved through discussion. Fourteen eligible studies were included. The synthesis examined intervention types (e.g., flipped classrooms, e-modules), outcomes (knowledge gain, engagement, satisfaction), and implementation barriers. Most studies reported improved post-test scores and high learner satisfaction with blended learning models.

**Results and conclusion:**

Most studies reported better retention and a preference for blended formats. Students appreciated flexibility, personalized learning, and collaborative opportunities. Reported barriers included poor internet connectivity, limited access to digital devices in rural areas, and low faculty confidence in digital teaching. Faculty acknowledged the benefits of BL but highlighted the need for structured methods and training. Policy-level support, investment in digital infrastructure, and integration of learning management systems (LMS) were recognized as enabling factors.

## Graphical Abstract

**Figure:**
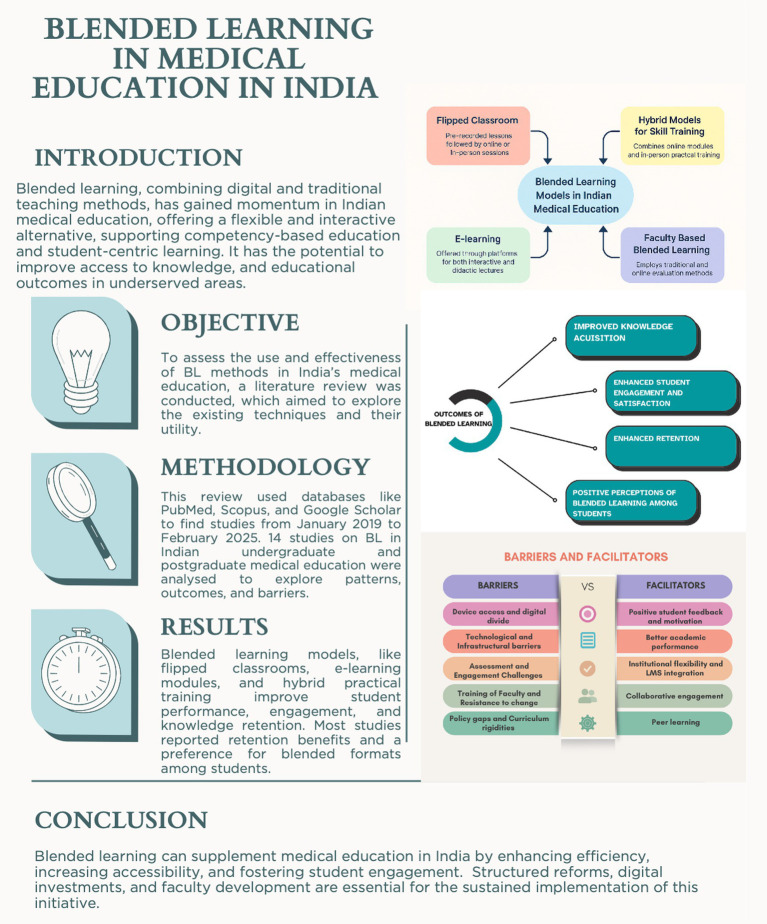

## 1 Introduction

Medical education in India has experienced a major transformation in recent decades. Conventionally, medical training has been imparted through face-to-face pedagogy, with students gaining knowledge through hands-on clinical sessions, in-person lectures, and practical training in hospitals and medical laboratories. With the advancement in technology and the pressing need for flexible learning mechanisms, blended learning (BL) has emerged as a promising approach to bridge the gaps in traditional and modern medical education (1).

The COVID-19 pandemic served as a major catalyst for educational reform, forcing an abrupt shift to online teaching and accelerating the adoption of technology-enabled learning across medical schools across the world (2). Consequently, BL has become a fulfilling medical paradigm combining in-person modalities with online learning, providing an alternative and presumably more enhanced medical education model (3). Given the unexpected scenario, which can also occur in the future, BL is a viable solution to fulfill educational needs under imposed restrictions (4).

Blended learning is an educational approach that combines online and in-person learning to enhance student engagement and facilitate knowledge retention. This model involves the integration of medical educational content in any digital format and allows students to use this content to enhance learning during in-person teaching methods like classroom teaching, hands-on clinical sessions, and/or laboratory discussions. Globally, BL has demonstrated potential to improve accessibility, support learner autonomy, and enhance knowledge retention through flexible, student-centered pedagogies. In a country like India, BL can be highly beneficial in improving flexibility, efficiency, and accessibility due to the high student-to-teacher ratio, limited resources in remote areas, and the need to cater to diverse learning needs. Digital resources such as e-learning modules, virtual simulations, and AI-led educational tools are being used to enhance the learning experience on the global platform. Hence, implementing BL in medical education in India aligns with global trends in medical pedagogy (5–7).

Despite these benefits, blended learning is not without its limitations. The digital divide remains a critical concern, with inequitable access to devices and reliable internet connectivity disproportionately affecting students in rural and semi-urban settings (6–9). Students adapting simultaneously to new content and unfamiliar delivery formats may experience increased cognitive load, potentially undermining learning efficiency. Maintaining motivation and self-regulation during asynchronous, self-paced online modules has also been identified as a recurring challenge, particularly among students who thrive in structured, face-to-face environments (10). Furthermore, the assessment of clinical and procedural skills through digital platforms remains inherently limited, as hands-on competency development requires physical presence and direct supervision (11). Finally, faculty workload, limited digital literacy, and resistance to pedagogical change represent significant implementation barriers, particularly in institutions where technology adoption is nascent (12). Acknowledging these limitations alongside BL’s considerable benefits is essential to justify the need for context-specific, evidence-based research on its implementation in Indian medical education.

Several factors support the need for implementing BL in medical education in India. With the rise in medical knowledge and advancements in healthcare technology, medical students must continually update their knowledge and skills. With the increasing use of telemedicine, AI, and electronic health records, exposure to digital learning prepares students for tech-enabled healthcare environments. Conventional in-person learning is, therefore, inadequate to keep up with the evolving nature of medicine. Blended learning ensures personalized learning, helping students stay updated with the latest findings in medical education. Implementing BL can benefit medical students from remote areas by providing them with access to the latest medical content and expertise from professionals (13).

Blended learning also encourages problem-solving, self-directed learning, and critical thinking, crucial elements for future medical professionals. Certain online components of BL or lessons, such as virtual patient simulations and online case discussions, enhance students’ decision-making and clinical reasoning skills during in-person encounters. The incorporation of online learning methods involves collaborative learning, including interactions with peers, online assessments, and discussion forums, which in turn enhances the educational experience for all students (3, 14).

Collaborative efforts are needed from medical experts, governments, and policymakers to address these barriers and ensure equitable access, including comprehensive training of medical teachers, enhanced infrastructure, and devising techniques to involve students efficiently in the online learning environment (15).

The National Medical Commission (NMC) is essential in approving educational methods and shaping the medical curriculum in India. Although online learning is widely accepted and BL continues to evolve across the globe in various other higher educational institutes, it needs to be explored how much the medical educational ecosystem in India is utilizing it. The NMC has emphasized the importance of adequately implementing BL modalities (16). Implementing BL models can be enhanced through meaningful collaborations between digital providers and medical institutes. The NMC guidelines further highlight the need for such collaborations (16). A national policy is also being developed with the goal of providing a structured framework to implement BL in institutes of higher education and ensure unmet needs, including training of medical experts, infrastructural needs, and technology requirements to ensure quality assurance and setting clear accreditation standards (17).

Presently, global reviews underscore the advantages of blended learning (BL) in health professions education; nevertheless, there is insufficient consolidated evidence regarding the implementation of BL within India’s distinct educational, technological, and policy contexts. To fill this gap, this narrative review looks at studies published between 2019 and 2025 that look at BL practices in Indian undergraduate and postgraduate medical education. The review seeks to (1) identify BL models presently employed in Indian medical institutions, (2) assess outcomes pertaining to knowledge acquisition, student engagement, and academic performance, and (3) examine contextual barriers and facilitating factors affecting implementation.

This review aims to inform educators, policymakers, curriculum developers, and institutions seeking to integrate BL effectively and sustainably within the evolving Indian medical education ecosystem by providing a comprehensive synthesis of recent empirical evidence.

## 2 Methodology

The review focused on identifying patterns in implementation strategies, learner outcomes, and system-level challenges across Indian medical institutions. This review adopted a structured narrative review approach and focused on identifying patterns in implementation strategies, learner outcomes and system-level challenges across Indian medical institutions. The research question was framed using the PICO framework: Population (Undergraduate and postgraduate medical students, interns, and faculty involved in medical education in India). Intervention (Blended learning strategies including flipped classrooms, e-learning modules, LMS-supported teaching, and hybrid practical training), Comparator (Traditional face-to-face teaching methods or conventional didactic lectures where applicable), and Outcomes (Knowledge acquisition, engagement, retention, academic performance, and learner perceptions). Specifically, literature was searched, studies were collated, and data were synthesized in this narrative review to address the following questions:

1. What are the BL methods currently being utilized?
2. What are the reported outcomes regarding knowledge acquisition, student engagement, and academic performance?
3. What are the implementation barriers and enablers unique to the Indian context?

### 2.1 Inclusion and exclusion criteria

Inclusion criteria:

- Research-based data driven studies (quantitative or mixed-method) focused on BL
- Studies evaluating undergraduate or postgraduate medical education in India
- Published between January 2019 and February 2025

Exclusion criteria:

- Reviews, editorials, letters, and commentaries
- Purely qualitative studies (Purely qualitative studies were excluded because this review aimed to assess measurable educational outcomes such as knowledge acquisition, test performance, and engagement metrics, which require quantitative or mixed-methods data)
- Studies with missing component of online or in-person teaching methods
- Research unrelated to medical education or outside the Indian context
- Studies in languages other than English

### 2.2 Search and screening process

To conduct a comprehensive literature search was conducted in four electronic databases, Scopus, Web of Science, PubMed and Google Scholar. The search strategy was developed iteratively and adapted for each database. Boolean operators and keyword combinations were used to capture variations of blended learning terminology. The search was performed in February and March 2025, targeting studies published in English between January 2019 and February 2025, to ensure that the latest findings are presented in the review. The key search terms used in the electronic database searches were ‘Blended learning’ OR ‘digital learning’ OR ‘e-learning’ OR ‘flipped classroom’ OR ‘online learning’ OR ‘hybrid learning’ AND ‘medical education’ OR ‘medical institute’ OR “health sciences education” AND ‘India’. These studies were evaluated through a three-step process to determine the exclusion and inclusion for the review: 1. Title screening for relevance; 2. Abstract review to assess eligibility; and 3. Full-text analysis of selected studies. After conducting the initial search and removing duplicates, abstracts were screened for relevance. Reference lists of the selected articles were also examined to find out any other relevant studies that the search strategy might have missed. Two independent reviewers then assessed and analyzed the full-text articles based on the inclusion and exclusion criteria. Any disagreement was resolved through discussion and consensus.

After conducting the initial search, we identified a total of 537 articles. After removing duplicates, 462 records were included for preliminary screening. The articles were selected based on the inclusion and exclusion criteria and abstracts were screened for relevance, of which 320 were excluded for not meeting inclusion criteria (e.g., not BL-focused, non-medical, or non-Indian context). The remaining full-text articles were assessed independently by two reviewers. A total of 102 articles were excluded at this stage due to reasons such as irrelevance to the research topic, access limitations (e.g., subscription-only), or because the studies were conducted outside India.

Additionally, reference lists of the selected articles were reviewed to find any other relevant studies. Finally, 14 studies met all criteria and were included in the review. To enhance methodological rigor, the included studies were subjected to a descriptive quality appraisal using adapted educational research quality indicators, including clarity of intervention, appropriateness of study design, validity of outcome measures, and completeness of reporting (Supplementary Table 1). Given the structured narrative review design and heterogeneity of included studies, a formal standardized risk-of-bias tool was not applied, and a formal meta-analysis was not feasible; however, methodological limitations were considered during narrative synthesis and a narrative approach was used which allowed integration of diverse findings into thematic categories for thematic analysis.

## 3 Results

Across the 14 included studies, the median sample size was 150 participants (range: 42–690). Most studies involved undergraduate MBBS students (78.5%), followed by faculty participants and medical interns. In terms of study design, 50% were cross-sectional surveys, 35.7% were interventional studies, and 14.3% used mixed methods approaches. Of the 14 studies, 8 (57.1%) employed pre/post-test designs, all of which reported statistically significant improvements in student performance (*p* < 0.05). The flipped classroom model was the most frequently used blended learning strategy (64.3%), followed by e-learning modules and LMS-based teaching tools (42.9%). Table 1 outlines the characteristics of these studies.

**Table 1:** Characteristics of the studies included in the review.

S no	Title of study	Author	Year	Sample size and population	Study site	Study type	Tool used	Findings
1.	Effectiveness of flipped classroom model in teaching histology for first-year MBBS students based on competency-based BL: An interventional study (32)	Sharmila Aristotle et al.	January 2021	150 students 1st year MBBS students	Tamil Nadu	Interventional study	Pre- and post-tests for each traditional & FCR session with MCQs. Feedback survey based on a 5-point Likert scale	Significant improvements in post-test scores compared to pre-tests (*p* < 0.0001) for both traditional and FCR methods. FCR yielded higher post-test scores than traditional lectures (*p* < 0.0001). Student feedback was overwhelmingly positive, with many indicating that FCR enhanced their competency in histology. The study concluded that FCR is an effective and well-received teaching method for histology education.
2.	Medical education during COVID-19 associated lockdown: Faculty and students’ perspective (26)	Subhangi Gupta et al.	February 2021	248—students, 23—faculty Undergraduate students and faculty members	Delhi, NCR	Cross-sectional study	Online questionnaire survey using Google form	Faculty and students appreciated the continuity through online learning. Challenges included technical issues, reduced practical exposure, and engagement difficulties. Both groups emphasized the need for improved digital infrastructure and blended approaches post-pandemic for effective medical training.
3.	Effectiveness of BL in radiological anatomy for first year undergraduate medical students (30)	Chitra Nagaraj et al.	April 2021	150 students 1st year undergraduate medical students	Karnataka	Prospective study	Pre- and post-tests and questionnaire to assess perceptions LMS TYRO	The intervention involved five modules, incorporating online presentations and self-assessment quizzes. A significant improvement in knowledge was observed, with pre-test and post-test scores increasing from 17 ± 5.5 to 26 ± 4.7.6 (*p* < 0.0001). Student feedback indicated a positive reception, with 77% appreciating the integration of radiology with anatomy, 73% finding the approach engaging, and 74% expressing interest in this teaching method. The study concluded that BL is an effective and well-received strategy for teaching radiological anatomy.
4.	E-learning in medical education: students’ experience, challenges and perspectives: a cross-sectional study in India (25)	Kabita Barua et al.	December 2021	537 students Undergraduate medical students	Delhi	Cross-sectional study	A semi-structured questionnaire distributed through Google forms	46.7% of students regularly attended online classes. Key facilitators included prior scheduling (88.6%), sharing study materials (84%), and interactive discussions (71.1%). 42.6% reported challenges in acquiring clinical skills through e-learning. Students preferred a blended approach, combining e-learning for theory with traditional methods for clinical training. The study underscores the importance of addressing technical barriers and enhancing instructor engagement to optimize e-learning in medical education.
5.	Flipped Classroom versus Traditional Didactic Classroom in Medical Teaching: A Comparative Study (31)	Milav H. Bhavsar et al.	March 2022	100 students 1st year MBBS students	Gujarat	Primary interventional educational study, crossover design	Two groups based on pseudo randomization created and subjected to FC & TDC methods in Module A and crossover of groups done in Module B. Both groups subjected to post-test after intervention.	FC significantly improved post-test scores compared to TDC (14.77 ± 2.16 vs. 12.16 ± 2.05, *p* < 0.05). Student feedback indicated a positive perception of FC, highlighting its effectiveness in enhancing learning outcomes. The study concluded that FC is a beneficial teaching method for medical education.
6.	Perceptions of Undergraduate Medical Students toward Online Learning in a Medical College in the National Capital Region (NCR), India (21)	Tuhina Shree et al.	June 2022	377 students 1st, 2nd & 3rd year undergraduate medical students	Uttar Pradesh	Cross-sectional study	Pre-designed, semi-structured questionnaire	The findings revealed that 54.9% of students preferred a BL approach, combining both online and offline methods. Challenges identified included lack of interaction with teachers (66.3%) and peers (57.6%), inadequate quiet spaces at home (44%), and poor internet connectivity (78.5%). Students expressed a positive outlook toward BL, though they were less enthusiastic about online learning compared to traditional classroom settings. The study suggests that integrating online education with conventional methods could enhance the learning experience for medical students.
7.	Flipped Classroom (FCR) as an Effective Teaching-Learning Module for a Large Classroom: A Mixed Method Approach (20)	Manpreet Kaur et al.	August 2022	170 students 1st year undergraduate MBBS studies	Delhi	Cross-sectional interventional study, mixed-method approach	Pre- and post-assessments, subjective feedback from students and teachers on a validated feedback survey	Pre- and post-assessments revealed significant improvement in student performance, particularly among low achievers. Feedback from both students and teachers indicated high satisfaction with FCR’s effectiveness and feasibility in large classrooms. Despite increased workload, FCR was deemed a valuable teaching-learning module.
8	Online assessment vs. Traditional assessment: perception of medical teachers in a tertiary level teaching hospital in South India (29)	Anitha Nancy et al.	September 2022	45 faculty Preclinical medical faculty	Puducherry	Cross-sectional survey	Google form using standard and validated questionnaires with Likert scale scoring	Findings indicated that while 96% of faculty agreed online assessments reduced paper correction workload, only 50% felt competent to handle them due to lack of training. Despite recognizing the efficiency of online assessments, nearly 40% believed they primarily evaluated cognitive knowledge. The study suggests a preference for a blended assessment approach, combining traditional methods with online assessments.
9.	E-learning and E-modules in medical education—A SOAR analysis using perception of undergraduate students (36)	Archana Prabu Kumar et al.	May 2023	690 students 1st year MBBS students and 1st year BDS students	Medical College in India	Longitudinal study	Two structured and validated questionnaires administered via MOODLE/hard copy	The study revealed that undergraduate medical students perceived e-learning and e-modules positively, highlighting enhanced accessibility, flexibility, and self-paced learning. Challenges included technical issues and reduced interaction. Overall, e-learning was seen as a valuable supplement to traditional education, with recommendations to improve engagement and infrastructure for better outcomes.
10.	The Role of the Flipped Classroom Method in Short-Term and Long-Term Retention Among Undergraduate Medical Students of Anatomy (33)	Payal Kasat et al.	September 2023	50 students 1st year undergraduate MBBS students	Medical teaching institute in India	Prospective, mixed methods study	Students subjected to traditional and FCR modules. Assessment after end of each module with formative assessment again after 2 months	The FC approach significantly enhanced short-term retention (p < 0.0001) and was found to be an effective and motivating tool for learning anatomical content.
11	The Impact of Patient-Centric Interactive E-Module in Pathology Among Medical Undergraduates (34)	Jayaprakash Venkatesan et al.	October 2023	60 students 2nd year undergraduate medical students	Puducherry	Mixed methods intervention study	Pre- and post-tests with MCQs using Google forms	Students understanding of pathology was significantly improved by patient-focused interactive e-modules. Because of their increased involvement, flexibility, and interactivity, the students favored e-modules. Compared to seminars, e-modules improved higher-order thinking. To increase comprehension and recall of pathology education, a BL paradigm that incorporates seminars and e-modules was proposed.
12	Effectiveness of the Flipped Classroom Teaching and Learning Method Among Underachievers in Physiology: Experience from a Tertiary Care Teaching Hospital (22)	L. Reshma Shireesha et al.	May 2024	100 students 1st year underachiever MBBS Students	Andhra Pradesh	Prospective, descriptive, cross-sectional study	Pre and post-test using Questionnaires quantified on a Likert scale	The results indicated that FC significantly improved students’ cognitive skills and analytical thinking, leading to better examination scores. FC promoted self-directed learning and peer-based interactions. The majority of students found FC to be an engaging and satisfactory learning experience. The study suggests that integrating FC with traditional teaching methods can enhance understanding of complex topics.
13	Comparative Study of the Flipped Classroom and Traditional Lecture Methods in Anatomy Teaching (35)	Shweta Jha et al.	July 2024	96 students First-phase undergraduate studies	Patna	Prospective interventional cross-over study	Pre- and post-tests by a pre-validated questionnaire quantified on a Likert scale	The results indicated that the FC method significantly improved student performance, particularly in regional anatomy, and enhanced engagement and critical thinking. Students expressed positive perceptions toward FC, highlighting its effectiveness in promoting active learning and clinical relevance. The study concluded that FC is a beneficial teaching strategy for anatomy education.
14	Perceived usefulness of a BL approach for skills training among medical interns: A pilot study (28)	Rashmi Ramachandran et al.	November 2024	42 interns Medical interns	Delhi	Mixed methods study	Online questionnaire based on 5-item Likert scale Focused group discussions (FGDs)	Medical interns perceived BL for skills training as highly useful, improving knowledge retention and practical competence. Participants valued the combination of online and hands-on sessions but suggested enhancements in content interactivity and scheduling flexibility to optimize learning experiences and outcomes.

### 3.1 BL models currently utilized in Indian medical education

**Figure 1:**
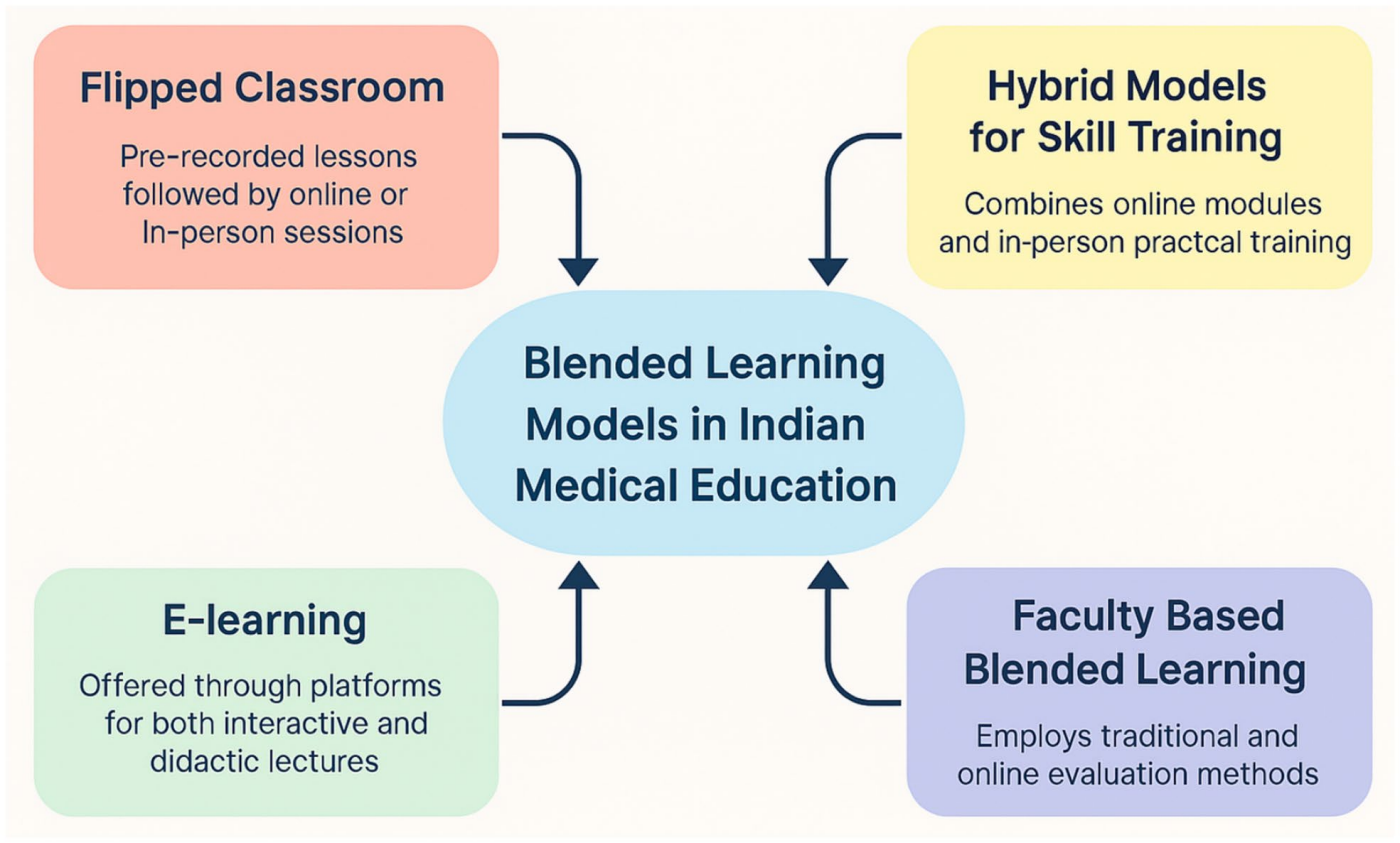
Conceptual diagram illustrating the four blended learning (BL) models currently utilized in Indian undergraduate and postgraduate medical education, as identified across the 14 included studies.

The studies describe how BL models have been applied in various ways within the Indian medical education system, incorporating in-person instruction with online assessments, flipped classroom methods, e-modules, and learning management systems. Figure 1 shows types of BL modalities utilized in Indian medical education.

#### 3.1.1 Flipped classroom

The flipped classroom was the most widely adopted BL approach, used in 9 out of 14 studies (64.3%). FC approaches were applied across disciplines, including anatomy, physiology, pathology, biochemistry, and histology. In this approach, students watched pre-recorded material before participating in online or in-person sessions for case-based learning and discussions. This enhanced students’ interest in and comprehension of the material (18, 19). It also improved retention and students’ learning abilities. Aristotle et al. reported significantly higher post-test scores in FC sessions compared with traditional lectures (*p* < 0.0001) (6). Bhavsar et al. demonstrated improved performance in FC vs. traditional didactic classroom (14.77 ± 2.16 vs. 12.16 ± 2.05; *p* < 0.05) (10). A study showed that mean post-test scores were higher than pre-test scores. (8.2 ± 1.0 vs. 5.5 ± 1.2) upon using a flipped classroom approach with a statistically significant enhancement in understanding. In this study, 85% of students agreed that the flipped classroom approach improved their learning experience, and 78% reported feeling more engaged (20). Kaur et al. found FC particularly beneficial for low achievers, enhancing participation and performance (21). Another study found that 56% of the participants agreed and 21% strongly agreed that the flipped classroom method improved the students’ understanding of the subject more effectively (22).

#### 3.1.2 E-learning

“E-learning functions as a key delivery component within blended learning models. In the studies reviewed, e-learning elements (including learning management systems, asynchronous modules, and online quizzes) were integrated with in-person instruction to form the blended format.” Six studies (42.9%) employed e-learning components, using learning management systems (LMS) TYRO, MOODLE, online quizzes, asynchronous modules, or interactive e-content, and Google Forms-based assessments and feedback tools. These resources supported both interactive learning modes and didactic lectures (23, 24). Barua et al. reported that 46.7% of students consistently attended online lectures and 88.6% were satisfied with pre-scheduled lectures (25). In another study, 88.3% of students felt that online classes were effective in helping them continue their education during the COVID-19 pandemic (26). Venkatesan et al. observed patient-centric interactive e-modules enhanced higher-order thinking and engagement (27).

#### 3.1.3 Hybrid models for skill training

One study evaluated a hybrid BL model for clinical skills training among medical interns a study evaluated a hybrid BL model for clinical skill training among medical interns. The study concluded that incorporating online modules with practical training sessions can lead to improved outcomes in medical training. Mean perceived importance scores were: 4.9 (SD 0.2) to 5.0 (SD 0) out of 5 across all skills and post-training confidence rates improved. 97.6% interns were confident in handwashing and gloving/gowning and 88.1% were confident in IV cannulation. Interns reported improved confidence, knowledge retention, and procedural competence. They recommended enhancing the interactivity and flexibility of online content (28).

#### 3.1.4 Faculty-based BL

The faculty-based BL model refers to an instructional format where faculty members serve as both designers and deliverers of blended content, integrating online assessments, digital feedback tools, and in-person teaching. In the study by Nancy et al. (29), preclinical faculty at a South Indian tertiary hospital used a combined approach of online Google Form-based assessments alongside traditional face-to-face teaching sessions, and reported that 96% of faculty members preferred online assessments due to their logistical ease; however, only 50% felt adequately trained to use online platforms. Faculty members acknowledged the need for training in digital pedagogy to effectively contribute to blended education modules.

### 3.2 Reported outcomes of blended learning in Indian medical education

**Figure 2:**
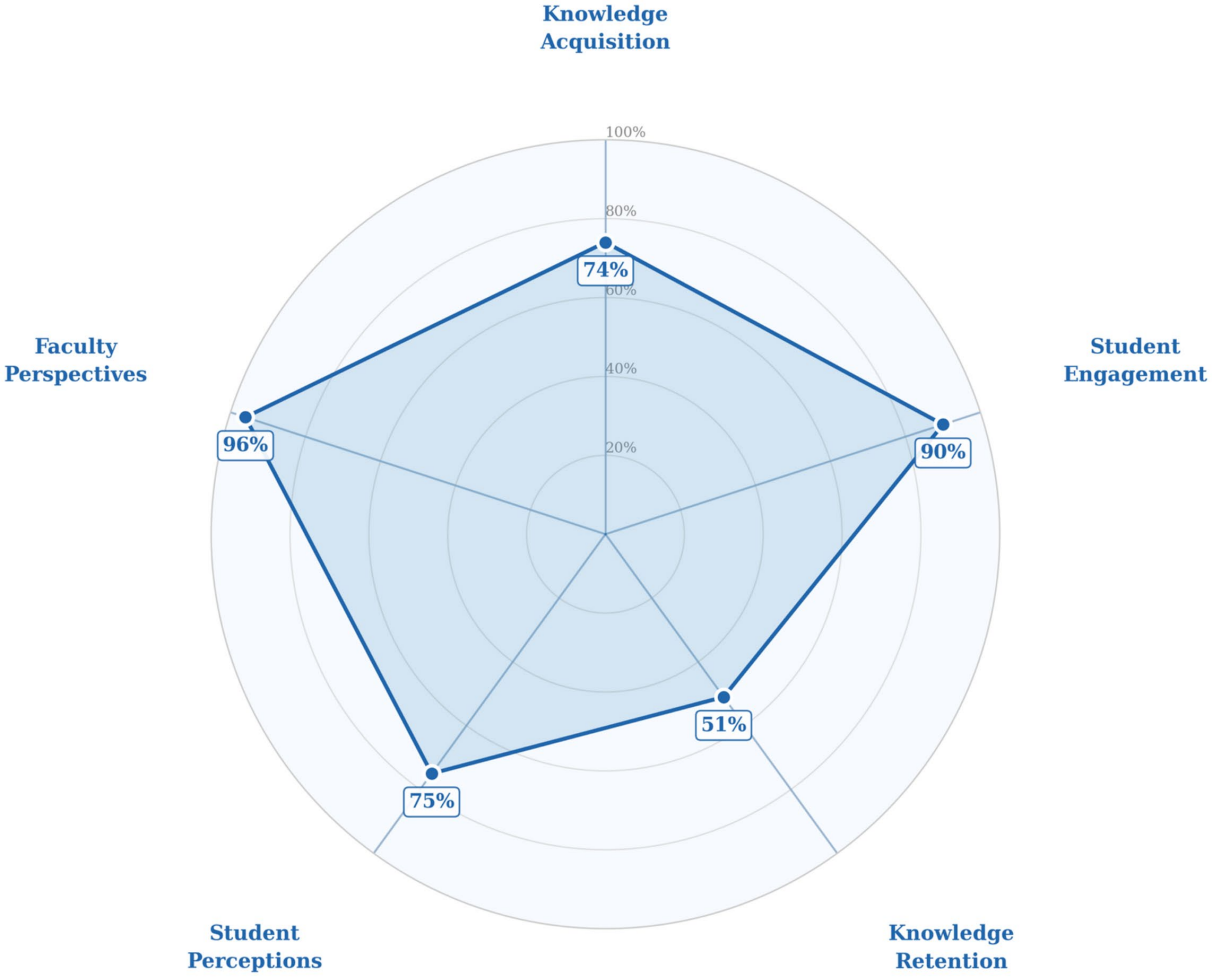
Radar chart depicting the impact of blended learning (BL) across five domains in Indian medical education (*n* = 14).

All 14 studies included in our review reported positive educational outcomes regarding BL techniques. Figure 2 shows the impact of blended learning on students learning and performance.

#### 3.2.1 Learning outcomes

Improved knowledge acquisition and better academic performance—In nearly all studies employing pre- and post-test designs, a statistically significant improvement in test scores was observed after the intervention. Consistent improvement in academic performance was also documented studies. In one of the studies conducted in radiological anatomy (30), the scores improved nearly 50% from 17 ± 5.5 to 26 ± 7.6 with a statistical significance (*p* < 0.0001), indicating effective knowledge gain through BL. Another study demonstrated considerably higher post-test results using the flipped classroom method in contrast to the traditional lectures (14.77 ± 2.16 vs. 12.16 ± 2.05, *p* < 0.05). A significant increase in test scores was observed, following the integration of blended modules providing strong empirical support for the pedagogical efficacy of BL (31).

Enhanced student engagement– Students showed strong acceptance of BL models such as the flipped classroom and e-learning models. A study reported that over 90% of students preferred the FC model for learning histology due to enhanced interactivity and comprehension (32). Another study found that the flipped classroom model promoted self-guided learning and collaboration among peers, especially among underachieving students (22).

Enhanced retention—Studies have shown an improvement in the retention power of students due to the implementation of BL models. Kasat et al. demonstrated short term [Traditional 31.88 ± 19.95 vs. Flipped 51.4 ± 14.09 (*p* < 0.0001)] and long term [Traditional 52.33 ± 22.1 vs. Flipped 59.0 ± 17.2 (*p* < 0.095)] retention gains at two-month follow-up (33). Retention benefits are attributed to repeated exposure, active learning and integration of theory and practical reasoning.

#### 3.2.2 Perceptual outcomes

Student Perceptions of Blended Models—Students expressed a favorable preference for BL over strictly online or conventional modes. Convenience, access to recorded knowledge, and enhanced conceptual clarity were among the benefits mentioned. A study conducted among undergraduate medical students in a medical institute in Uttar Pradesh reported that 54.9% of students preferred a BL approach over a completely online one (21). In many studies, 73–77% of students found blended models to be more engaging and effective (34, 35). In another study, students appreciated the flexibility and accessibility of the BL techniques (36).

Faculty Perspectives—Two studies evaluated faculty perceptions on BL and reported that 96% of faculty members preferred online assessments due to their logistical ease, but only 50% were competent enough to use them due to a lack of training. Faculty members acknowledged the need for training in digital pedagogy to effectively contribute to blended education modules (29). In a different study, 65.2% of faculty members supported incorporating online teaching modules into the current curriculum, with 69.6% suggesting a mix of 70% traditional to 30% online class ratio distribution (26).

### 3.3 BL implementation barriers

The incorporation of BL into Indian medical education, while promising, faces particular challenges. The key barriers that emerged in our review studies were as follows.

#### 3.3.1 Technological and infrastructural barriers

Lack of adequate digital infrastructure emerged as a significant barrier across studies, especially in semi-urban and rural areas. According to a study, 78.5% of students lacked adequate internet connectivity, and 44% of students lacked a conducive study space at home (21). According to another study, 42.6% of students experienced difficulty learning clinical skills online, suggesting a disconnect between theory and practice (25).

#### 3.3.2 Device access and digital divide

The digital divide was a recurring challenge. The uneven access to digital devices was clearly demonstrated in our review studies. Students from resource-limited settings in rural areas struggled to access laptops, smartphones, and reliable data services, which hindered their participation in the online modules (21, 25, 26).

#### 3.3.3 Training of faculty and resistance to change

Limited training in pedagogy and initial resistance to non-traditional teaching methods also emerged as a barrier, even though 96% of faculty preferred online assessments due to logistical ease, only 50% were competent enough to use them due to a lack of training (29).

#### 3.3.4 Assessment and engagement challenges

There were barriers about the assessment of students and their adequate engagement. In a study, faculty expressed concerns about the effectiveness of online evaluations, as they felt that digital tools only assess surface-level cognitive domains (29). In another study, students reported reduced interaction with peers (57.6%) and teachers (66.3%), indicating that intellectual and social engagement was lacking in purely digital setups.

#### 3.3.5 Policy gaps and curriculum rigidities

Despite a growing acceptance, BL has not yet been consistently included in the national curriculum. Our included studies highlighted a lack of the flexibility required for integrating BL due to structural inertia, including rigid institutional schedules and examination systems (26, 28).

### 3.4 Enablers of BL implementation

#### 3.4.1 Strong student motivation and positive feedback

In most of our studies, a high level of satisfaction was reported by students with the integration of the BL formats. In studies employing the flipped classroom approach, over 70% of students expressed that the flipped classroom methods improved their understanding, engagement and retention of core concepts (32, 33, 35). In another study, more than 70% of students appreciated the integration of BL methods for teaching radiological anatomy (30, 31).

#### 3.4.2 Institutional flexibility and LMS integration

Studies showed that institutes implemented learning management systems like MOODLE and TYRO, making it possible to distribute tests, feedback loops and modules in a systematic way (30, 36). These platforms assisted in standardizing content and facilitate asynchronous access, addressing the diversity in student learning styles.

#### 3.4.3 Collaborative engagement and peer learning

According to studies, the flipped classroom approach promoted more self-directed learning and engagement of peers. As per a study, the collaborative and interactive character of the BL format improved performance and satisfaction in the underachieving students (22).

**Figure 3:**
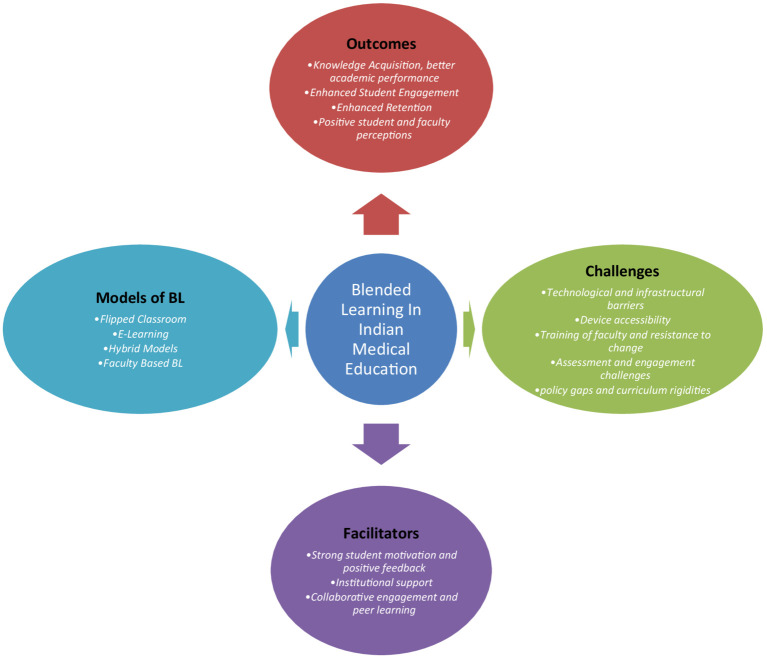
Conceptual map illustrating the components of BL in Indian medical education.

Figure 3 illustrates a concept map outlining the components of blended learning within the current Indian medical education system.

## 4 Discussion

This narrative review synthesizes evidence from 14 studies examining the implementation and outcomes of blended learning (BL) in Indian medical education. Across diverse institutional settings, subjects, and learner groups, BL consistently enhanced student performance, engagement, and knowledge retention. These findings reinforce global evidence demonstrating that BL can be a transformative pedagogical strategy in health professions education.

The findings of this review can be interpreted through several established educational frameworks. Constructivist Learning Theory (Vygotsky) (37) posits that learning is a socially mediated process shaped by collaborative interaction within the zone of proximal development. This directly underpins the effectiveness of the flipped classroom model observed across nine of the 14 included studies, wherein peer discussions, case-based learning, and in-person active sessions build upon independently acquired pre-class knowledge, fostering deeper conceptual understanding and collaborative skill development. The Adult Learning Theory proposed by Knowles et al. (38)—emphasizes that adult learners are self-directed, intrinsically motivated, and learn most effectively through problem-centered approaches that respect their autonomy and prior experience. This aligns closely with BL’s self-paced online components and competency-based instructional design, and is especially pertinent for the postgraduate trainees and medical interns included in this review, who demonstrated high levels of motivation and satisfaction with flexible BL formats. The Community of Inquiry (CoI) Framework (39), developed by Garrison, Anderson and Archer, proposes that meaningful educational experiences in blended and online environments emerge from the interaction of three presences: cognitive presence, social presence, and teaching presence. This framework directly explains the engagement inconsistency observed in this review—studies employing structured FC-based BL, where all three presences were sustained through purposive instructional design, reported high engagement, while studies conducted during COVID-19 lockdowns, where social and teaching presence were diminished in fully online formats, reported reduced student–teacher and peer interaction. Together, these three frameworks provide a coherent theoretical basis for understanding why BL yields stronger outcomes when its online components are purposively designed to complement, rather than replace, face-to-face learning.

Knowles et al. (38) BL is a revolutionary educational strategy that purposefully combines online and in-person learning experiences, utilizing technology to enhance student learning outcomes and foster student-centered environments (40). By moving the focus from teaching to learning- shifting from a teacher-centered to a student-centered approach- this method enhances students’ enthusiasm and engagement in the learning process, while also fostering greater dedication and tenacity (41). It makes it easier for students to interact with teachers and peers in virtual and real-world settings by fostering the growth of a digital community (42). One of the primary features of BL is that it combines face-to-face clinical training with online resources, such as video demonstrations, virtual simulations, and LMS-based assessments (43, 44) and it has capacity to support a variety of learning preferences and styles by providing a wide range of educational resources and activities. According to Dziuban et al. (45) BL creates a synergistic learning environment that maximizes knowledge acquisition and skill development by fostering a dynamic interaction between cutting-edge technology and classic pedagogical methods (45).

In the included studies, various educational components were employed, including pre- and post-test assessments to evaluate learning, interactive learning modules, structured feedback surveys using a Likert scale, and focused group discussions. Majority of the studies included in our review (74%) used the flipped classroom teaching approach or a blended teaching method, and nearly 50% of the studies utilized digital modules and learning management systems for assessment and delivery of medical education.

Across the studies, BL was associated with significant improvements in students’ academic performance and satisfaction. Studies by Bhavsar et al. (31) and Nagaraj et al. (30) demonstrated statistically significant gains in post-test scores among students exposed to blended approaches compared to traditional didactic methods. It aligns with studies from Turkey (Başer & Şahin) (27) and Pakistan (Ali et al.) (46), which showed measurable gains in student understanding and satisfaction using flipped and hybrid models in interprofessional and emergency care training, respectively.

Long-term retention—often overlooked in medical pedagogical research—was examined in several studies and demonstrated robust improvements with BL. Students exposed to repeated reinforcement through videos, quizzes, and application-based discussions retained knowledge more effectively than those taught using traditional methods alone. This aligns with memory and cognition theories suggesting that multimodal, active, and spaced learning significantly enhances long-term retention. Similarly, a study from the United States by Frontino et al. (47) showed that simulation-rich hybrid learning environments promote long-term competency development in pediatric care.

A critical strength of BL identified in this review is its capacity to enhance learner engagement. Students valued the flexibility, interactivity, and personalization offered by BL approaches. Nine out of the 14 studies used the flipped classroom model, which encouraged critical thinking, peer interaction, and active learning. A study conducted among undergraduate students by Kasat et al. (33) found that flipped classrooms allow students to review material at their own pace before taking part in in-person discussions. This study also supported this finding through structured post-tests and follow-up assessments, which demonstrated retained knowledge over several weeks. This finding aligns with research conducted by Long et al. (48) and Sletten (49) in Western settings, where flipped classrooms have consistently yielded improved comprehension and increased learner autonomy. In India, flipped classroom models have led to statistically significant gains in post-test performance and have been preferred over didactic lectures by more than 70% of students, as observed in studies by Aristotle et al. and Shireesha et al. (22, 32). These patterns mirror global studies emphasizing student-centered learning, such as a motivational ARCS-V model-based program in Turkey (27), which also improved participation and long-term retention.

The apparent contradiction in engagement findings can be explained by context and BL model type. Studies reporting enhanced engagement predominantly used structured flipped classroom models with active in-person components, where online content supplemented face-to-face discussion. In contrast, reduced interaction was reported in studies conducted during COVID-19 lockdowns or in fully online formats, where in-person sessions were absent. This suggests that BL enhances engagement when the in-person component is preserved and purposively designed, but engagement may suffer when online delivery replaces rather than supplements classroom interaction. Future BL implementations should ensure a meaningful face-to-face component to sustain student–teacher and peer interactions.

Globally, faculty development remains a critical component in ensuring the success of blended approaches. According to a study done by Shrivastava et al. (3), medical faculty initially showed a reluctance to adopt new technology and reported difficulties using online tests and engagement tools. However, as Singh et al. (24) noted in their study, many people adjusted to using quizzes, discussion boards, and analytics dashboards effectively with the proper training and institutional support. Due to automated assessments, faculty reported a reduced workload in grading, and they valued the analytics that highlighted learning gaps. In a study conducted by Nancy et al. (29) 96% of Indian faculty members preferred online assessments for logistical ease, however, consistent with global challenges, faculty reported limited confidence in creating and delivering digital content. This gap highlights the need for institutional investment in faculty development programs focusing on: digital pedagogy, online assessment design, instructional technology, and student engagement techniques in virtual settings. This gap is consistent globally, with educators in the UK and UAE citing insufficient digital literacy as a significant hurdle to effective blended instruction (Mohiyeddini & Loftus) (50).

Besides functioning as a temporary solution during the COVID-19 pandemic, BL has evolved into a strategic educational innovation in India and will expectantly bring about long-term reforms. BL offers a scalable and equitable solution in a nation like India, where medical schools differ significantly in terms of faculty availability and infrastructure.

Blended models benefit learners by combining self-paced online learning with in-person opportunities for clarification and application. Such models align well with adult learning principles and contemporary medical education frameworks, shifting from teacher-centered instruction to student-centered, competency-based learning. Particularly noteworthy is the advantage observed among underachieving students, who displayed higher engagement and improved conceptual understanding when exposed to FC-based BL approaches. This reinforces BL’s potential to narrow achievement gaps within heterogeneous learning groups.

Despite its promise, the implementation of BL in Indian settings faces systemic and infrastructural barriers. Common issues included inadequate institutional support, unstable internet access in semi-urban regions, and a lack of faculty training. Faculty members expressed concerns that online tests are limited to lower-order cognitive evaluations, suggesting the need for more comprehensive digital pedagogical training. Technological barriers, including poor internet connectivity, limited access to devices, and infrastructural deficits, emerged as primary constraints in both Indian and global low- and middle-income country (LMIC) settings. In India, over 78% of students reported connectivity issues, as found in a study by Shree et al. (21). The absence of standardized digital infrastructure and faculty development programs, however, remains a persistent issue. The digital divide widened because urban institutions were more prepared than rural ones (25). Similar findings were observed in countries such as Jordan and parts of sub-Saharan Africa, which had reported infrastructural and connectivity issues as depicted in the study by Mohamed et al. (51). These findings highlight the importance of investing in digital equity and national infrastructure development.

Encouragingly, the National Medical Commission (NMC) in India has begun advocating for structured BL implementation within the Competency-Based Medical Education (CBME) framework. Yet, standardized policy enforcement and curriculum integration are still evolving. These policy-level endorsements, noted in several studies reviewed, call for collaboration between educational technology providers and academic institutions. In contrast, developed countries have integrated hybrid models into their evaluation and accreditation processes, offering more transparent pathways for sustainability (17).

This review adds to the literature by identifying India-specific enablers—like high student motivation, LMS platform availability, and measurable performance improvements—that can help expand BL more widely. It also offers actionable insights for policymakers and institutional leaders by highlighting infrastructural and faculty-related barriers.

Based on the synthesis of findings, several implications emerge:

- Infrastructure Investment: priority investments should target reliable broadband internet in rural and semi-urban campuses, institutional provision of laptops or tablets for students with limited device access, and procurement/licensing of LMS platforms (e.g., MOODLE, TYRO) across all medical colleges.
- Institutional faculty training programs for successful large-scale implementation: Structured faculty development programs covering digital pedagogy, online assessment design (beyond surface-level MCQs to include higher-order cognitive evaluation), LMS platform use, and virtual student engagement strategies should be mandated institution-wide before BL rollout.
- Curriculum Redesign: Based on faculty preference data (69.6% recommending a 70:30 traditional-to-online ratio), curricula should allocate approximately 30% of instructional time to online components—including pre-class asynchronous videos, digital quizzes, and e-modules—while retaining 70% for in-person clinical and applied learning. Skills-based sessions should remain predominantly face-to-face
- Cross-institutional partnerships with ed-tech platforms; BL curriculum modules: Partnerships with national ed-tech platforms and institutional e-learning portals can provide pre-built, quality-assured medical content modules, reducing individual faculty workload and enabling standardization across institutions with varying resource levels.
- A strong national policy for digital medical education: The National Medical Commission (NMC) should develop binding BL integration guidelines within the CBME framework, specifying minimum digital infrastructure standards, mandatory faculty competency thresholds, assessment modality proportions, and quality assurance mechanisms to ensure equitable and consistent BL delivery across all accredited medical colleges.

India’s path toward blended medical education is comparable to that of other low- and middle-income nations, where BL is being increasingly adopted as a bridge between traditional approaches and digital transformations. Unlike high-resource settings, India must address both sociocultural resistance to pedagogical change and digital divides simultaneously. Reliable internet access, device availability, and LMS implementation are foundational to successful BL adoption, especially in remote or under-resourced institutions. Medical curricula should incorporate BL components systematically rather than as temporary or supplementary interventions. This includes scheduled online modules, digital assessments aligned with competencies and hybrid approaches for skills-based teaching. Findings from Indian contexts align with international evidence showing the effectiveness of BL in improving learning outcomes and student engagement, as reported in studies by Liu et al. (52) and Vallée et al. (12). However, context-sensitive models that integrate low-tech and high-tech tools are necessary to address the challenges faced by India, especially its linguistic and infrastructure diversity. India’s deployment of tools like Moodle and Tyro aligns with global best practices (30, 36). International institutions are increasingly integrating AI-based adaptive learning, gamification, and augmented reality (AR) to create immersive hybrid experiences—areas where Indian models are still in their nascent stages.

In Indian medical education, BL has a potentially bright future with the implementation of policy-level reforms that can support standardization, ensure quality, and encourage collaborations between medical colleges and educational technology partners. Strong hybrid teaching methods that combine online and offline best practices are being called for. It is anticipated that innovations such as virtual dissection laboratories, AI-driven adaptive learning, and integrated digital assessment tools will enhance the effectiveness and scalability of BL in India’s diverse educational environments.

While global reviews have documented the advantages of BL in medical education, few have examined its adoption specifically within India’s diverse educational contexts. This review addresses that gap by synthesizing recent empirical studies and identifying contextual nuances—such as the digital divide, variability in institutional readiness, and specific faculty training needs—that influence BL implementation in India.

Future research should focus on multi-institutional trials evaluating long-term knowledge retention, longitudinal studies tracking clinical competency outcomes beyond knowledge test scores, such as OSCE performance and clinical reasoning, and cost-effectiveness of blended learning models in Indian medical education. Further studies are also needed to address equity issues related to digital access and infrastructure disparities.

## 5 Strengths

This review offers a comprehensive synthesis of 14 recent empirical studies from diverse regions of India, ensuring that the findings are both context-specific and up to date, particularly in the post-COVID era. By including studies involving undergraduate students, postgraduate trainees, and faculty members, it captures multiple perspectives on blended learning and provides a holistic understanding of its impact. A key strength lies in its focus on educational outcomes beyond knowledge acquisition, encompassing student engagement, retention, and faculty viewpoints, which enrich the analysis. Furthermore, the review highlights barriers such as infrastructural gaps and limited faculty preparedness, while also identifying enabling factors like policy support and learning management systems. This dual emphasis on challenges and facilitators enhances the practical value of the findings for educators, institutions, and policymakers aiming to implement blended learning sustainably in Indian medical education.

## 6 Limitations

This review may have overlooked significant regional literature in local languages because it only included studies published in English. Although a descriptive quality appraisal was conducted, the absence of a formal standardized risk-of-bias assessment tool may limit the strength of methodological inferences. The included studies are geographically dispersed, with a concentration of research in South India and urban academic institutions, excluding remote or tribal regions where implementation challenges may differ, despite spanning multiple Indian states. Being a narrative review rather than a systematic review or meta-analysis, the synthesis is interpretative and descriptive. Qualitative insights that could have enhanced our understanding of the experiences of educators and learners were excluded due to initial criteria, despite providing rich user experiences. The implicit presumption that all institutions possess a baseline level of digital infrastructure and literacy may not accurately represent the situation in medical colleges with lower funding. Furthermore, one of the 14 included studies (Study 14: Ramachandran et al., 2024) was co-authored by members of the current review team (AR, RR). To minimize the risk of bias, this study was assessed using the same quality appraisal criteria applied to all other included studies, and an independent reviewer not involved as an author of Study 14 was responsible for its data extraction and appraisal. Readers should interpret findings attributed to this study with this potential conflict in mind.

## 7 Conclusion

In Indian medical education, BL is a revolutionary strategy that effectively bridges the gap between traditional teaching methods and technological advancements. Its beneficial effects on promoting active learning, improving accessibility, knowledge acquisition, learner engagement, and academic performance across a range of subjects, including anatomy, physiology, pathology, and skills-based modules, as well as adhering to global educational trends, are continuously highlighted by the narrative synthesis of recent studies.

Although BL integration is still in its early stages in India, it has shown promise in promoting equal opportunity to access high-quality education, particularly for students in rural or resource-constrained areas. Furthermore, it complements the objectives of competency-based medical education as outlined by the National Medical Commission and aligns with global trends in health sciences education.

While students benefit from flexibility, personalization, and enhanced conceptual clarity, faculty members are also becoming increasingly aware of the advantages of digital resources for instruction and evaluation. Successful implementation is challenging, however, due to issues such as inadequate digital infrastructure, gaps in faculty training, and limitations related to assessment, particularly in settings with limited resources. However, a diversified strategy is required to sustain and grow BL in India. Improved digital access in rural areas, learner support systems, faculty development, institutional preparedness, and policy-level guidance that integrates blended modules into national medical curricula are all essential enablers for successful implementation. Policymakers, educators, and technologists must collaborate to transition from pilot projects to scalable educational reforms. BL has the potential to democratize and modernize medical education in India by providing aspiring health professionals with both clinical proficiency and digital competence, provided that structured reforms and stakeholder alignment are implemented.

## Data Availability

Publicly available datasets were analyzed in this study PubMed/Google Scholar and are included in the article/Supplementary material, further inquiries can be directed to the corresponding author/s.
