# Supplementary material for: Blended learning in medical education in India—a literature-based analysis and description
Source: Front Med (Lausanne). 2026 Apr 30;13:1814270. doi: 10.3389/fmed.2026.1814270 (PMC13171311; doi:10.3389/fmed.2026.1814270)
Supplement: Supplementary file 1 [file Table_1.DOCX]

# Supplementary Table 1: Methodological Quality Appraisal of Included Studies

| S. No. | Author (Year) | Study Design | Sample Size | Clarity of BL Intervention | Outcome Measures Validity | Study Design Rigor | Bias Risk | Overall Quality |
| --- | --- | --- | --- | --- | --- | --- | --- | --- |
| 1 | **Aristotle et al. (2021)** | Interventional | 150 | Clear | Valid | High | Low | **High** |
| 2 | **Gupta et al. (2021)** | Cross-sectional | 271 | Moderate | Self-reported | Moderate | Moderate | **Moderate** |
| 3 | **Nagaraj et al. (2021)** | Prospective | 150 | Clear | Pre-post | High | Low | **High** |
| 4 | **Barua et al. (2021)** | Cross-sectional | 537 | Moderate | Questionnaire | Moderate | Moderate | **Moderate** |
| 5 | **Bhavsar et al. (2022)** | Interventional | 100 | Clear | Post-test | High | Low | **High** |
| 6 | **Shree et al. (2022)** | Cross-sectional | 377 | Moderate | Self-reported | Moderate | Moderate | **Moderate** |
| 7 | **Kaur et al. (2022)** | Mixed-method | 170 | Clear | Pre-post + feedback | High | Low | **High** |
| 8 | **Nancy et al. (2022)** | Cross-sectional | 45 | Moderate | Survey | Moderate | Moderate | **Moderate** |
| 9 | **Kumar et al. (2023)** | Longitudinal | 690 | Clear | Validated tools | High | Low | **High** |
| 10 | **Kasat et al. (2023)** | Mixed-method | 50 | Clear | Pre-post + follow-up | High | Low | **High** |
| 11 | **Venkatesan et al. (2023)** | Mixed-method | 60 | Clear | Pre-post | High | Low | **High** |
| 12 | **Shireesha et al. (2024)** | Prospective | 100 | Clear | Questionnaire | Moderate | Moderate | **Moderate** |
| 13 | **Jha et al. (2024)** | Interventional | 96 | Clear | Pre-post | High | Low | **High** |
| 14 | **Ramachandran et al. (2024)** | Mixed-method | 42 | Clear | Survey + FGD | Moderate | Moderate | **Moderate** |
